# The link between chronic rhinosinusitis and asthma: A questionnaire-based study

**DOI:** 10.1097/MD.0000000000004294

**Published:** 2016-08-07

**Authors:** Chien-Chia Huang, Chun-Hua Wang, Chia-Hsiang Fu, Chi-Che Huang, Po-Hung Chang, I-Wei Chen, Ta-Jen Lee

**Affiliations:** a Division of Rhinology, Department of Otolaryngology, Chang Gung Memorial Hospital; b Graduate Institute of Clinical Medical Sciences, College of Medicine; c Department of Thoracic Medicine, Chang Gung Memorial Hospital, Chang Gung University, Taoyuan, Taiwan.

**Keywords:** airway, asthma, chronic rhinosinusitis, quality of life, questionnaire

## Abstract

Treatments for chronic rhinosinusitis (CRS) and asthma can affect both conditions, based on the united airway concept. This study aimed to evaluate the link between CRS and asthma, based on disease-specific quality of life measures.

We performed a prospective cohort study to investigate the correlations between results from CRS- and asthma-specific questionnaires. Thirty-two patients with asthma and CRS were evaluated before and after undergoing nasal surgery at a tertiary medical center.

There were significant correlations between the results from the Asthma Control Test (ACT) and the Sino-Nasal Outcome Test-22, as well as between the results of the ACT and Rhinoconjunctivitis Quality of Life Questionnaire, at both the preoperative and 3-month postoperative evaluations (P < 0.01). Moreover, nasal surgery improved the sinonasal symptoms, asthma control, and pulmonary function (P < 0.01).

Increasingly severe sinonasal symptoms of CRS were associated with poor asthma control. Therefore, CRS and asthma should be considered and treated as common airway diseases.

## 1 Introduction

Chronic rhinosinusitis (CRS) is an advanced and long-standing inflammatory condition that affects the mucosa of the nose and sinuses.^[1]^ In this context, CRS and asthma are viewed as 2 manifestations of a single pathological process, based on the united airways concept.^[2,3]^ In addition, previous studies have identified cross-sectional and longitudinal associations between CRS and asthma.^[4–8]^ Furthermore, there is a high prevalence of CRS among patients with asthma, and the presence of CRS is associated with poor asthma outcomes.^[9–12]^ Moreover, comorbid asthma is an important risk factor for resistance to therapeutic interventions for CRS, such as endoscopic sinus surgery (ESS).^[13,14]^ Compared to patients who do not have asthma, patients with asthma and CRS have poorer outcomes, less quality of life (QoL) improvement, and a higher rate of revision surgery after ESS.^[15,16]^

During recent decades, patient-reported outcomes have been used to evaluate disease status, as these measures reflect the patient's perspective in the symptoms and effects that they experience.^[17,18]^ Thus, various CRS- and asthma-specific questionnaires have been introduced to measure disease severity and control over time. These questionnaires include the Sino-Nasal Outcome Test-22 (SNOT-22),^[19,20]^ the Rhinoconjunctivitis Quality of Life Questionnaire (RQLQ),^[21]^ and the Asthma Control Test (ACT).^[22,23]^

The SNOT-22 and RQLQ tools provide validated self-reported measures of symptom severity and health-related QoL among patients with sinonasal conditions.^[21,24]^ The SNOT-22 tool covers various symptoms, physical problems, functional limitations, and emotional consequences of having a sinonasal disorder.^[19]^ The RQLQ is a 28-item tool that evaluates 7 domains, which include activity limitations, sleep problems, nose symptoms, eye symptoms, nonnose/eye symptoms, practical problems, and emotional function.^[21]^ The SNOT-22 and RQLQ tools have already been adopted by many clinicians to assess CRS status and treatment outcomes (including after surgery).^[25–27]^ The ACT consists of 5 items regarding asthma control: activity limitations, shortness of breath, waking up because of asthma symptoms, use of asthma relief medication, and a global evaluation of control.^[23]^ The ACT items evaluate symptoms that were experienced during the last 4 weeks, and are scored from 1 to 5. The total score is used to indicate perfectly controlled asthma (25 points), well-controlled asthma (20–24 points), or poorly controlled asthma (≤19 points).^[28]^

To the best of our knowledge, no studies have evaluated the link between CRS and asthma based on changes in self-reported disease-specific QoL. Thus, this study aimed to investigate the correlations between these self-reported measures of QoL among patients with asthma and CRS before and after nasal surgery.

## 2 Materials and methods

### 2.1 Patients

This study's protocol was approved by the institutional review board of Chang Gung Memorial Hospital, and all patients provided their informed consent for participation and use of their clinical data. We prospectively enrolled consecutive patients with asthma and CRS who were being treated in the Thoracic and Otolaryngology Departments between August 2013 and July 2015. The inclusion criteria were patients with asthma who fulfilled the diagnostic criteria of the Global Initiative for Asthma (GINA) guidelines,^[29]^ had CRS that was diagnosed based on the European Position Paper on Rhinosinusitis and Nasal Polyps criteria,^[30]^ had failed 3 months of medical treatment (e.g., intranasal corticosteroids, antihistamines, and/or broad-spectrum oral antibiotics), and planned to undergo ESS for CRS. All patients’ asthma had been treated based on the GINA guidelines for ≥6 months before the surgery to achieve relatively stable control, which allowed them to undergo nasal surgery under general anesthesia. We excluded patients with major medical disorders, such as diabetes, nephrotic diseases, autoimmune disorders, immunodeficiency, malignancy, and other chronic illnesses.

### 2.2 Objective and disease-specific quality of life measurements

All patients underwent an endoscopic nasal examination and sinus computed tomography (CT) before surgery. The CT data were evaluated before the surgery by 2 independent senior rhinologists (CCH and CHF) using a Lund–Mackay CT scoring system.^[31]^ Each sinus was assigned a score between 0 (completely aerated) and 2 (completely occluded). The osteomeatal complex was scored as either 0 or 2, based on a similar methodology. The CT score for each patient ranged from 0 to 24. We also performed preoperative and 3-month postoperative pulmonary function tests, which evaluated forced vital capacity (FVC), forced expiratory volume in 1 s (FEV_1_), and the FEV_1_/FVC ratio. All participants completed the ACT, SNOT-22, and RQLQ questionnaires to evaluate their asthma control and sinonasal symptoms before and 3 months after nasal surgery.

### 2.3 Statistical analyses

Data were reported as mean ± standard error, and were analyzed using GraphPad Prism software (version 5; GraphPad Prism Software, Inc., San Diego, CA). Categorical variables were compared using the Chi-square test or Fisher exact test, as appropriate. Continuous variables were compared using the Wilcoxon signed-rank test. Correlations between 2 items were evaluated using Spearman correlation coefficient. A *P*-value of <0.05 was considered statistically significant.

## 3 Results

### 3.1 Clinical characteristics of the participants

**Figure 1:**
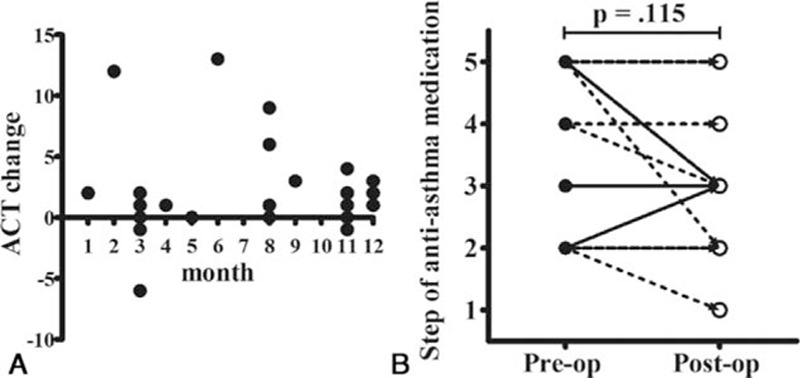
Patients underwent nasal surgery during nearly all months of the year. The Asthma Control Test (ACT) changes were not associated with the seasons (A), and most patients kept the same antiasthma medication over the course of the study (B). Asthma was treated according to the stepwise approach of the Global Initiative for Asthma guidelines29. Post-op = postoperative, pre-op = preoperative.

Table 1 shows the participants’ clinical characteristics. Twenty-two patients exhibited atopy; all of these patients were allergic to perennial allergens (the most common allergens were *Dermatophagoides pteronyssinus*, *Dermatophagoides farinae*, house dust, and cockroach), while 2 of these patients were also allergic to pollen allergens (eucalyptus and Bermuda grass). None of the patients were sensitive to pollen allergens alone. Patients underwent nasal surgery during nearly all months of the year, and no seasonal variations were observed in the outcomes (Fig. 1A).

**Table 1:**
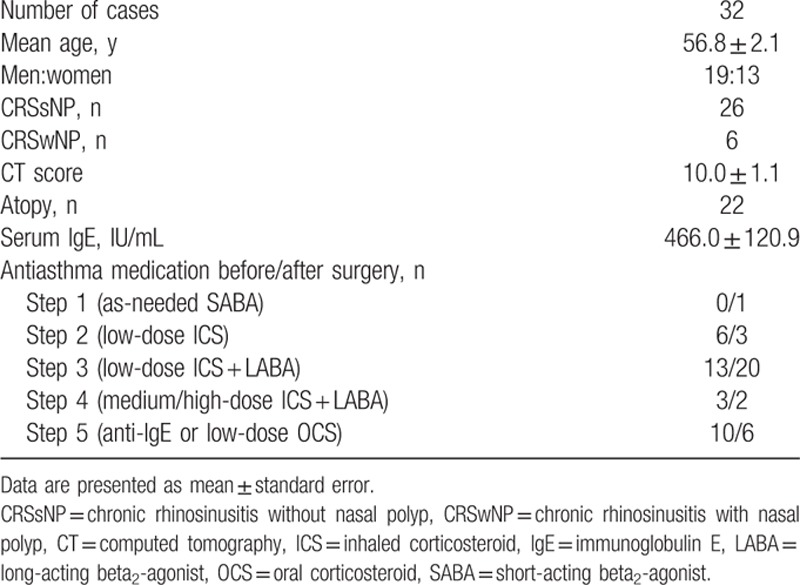
Clinical characteristics of the participants.

Most patients maintained the same antiasthma medication throughout the study (Fig. 1B). Six patients were shifted to less intense medication (their ACT improvements were 2, 0, −1, 1, 13, and 0) and three patients were shifted to more intense medication (their ACT improvements were 3, 4, and 0). The ACT improvements were not associated with medication change in these cases.

### 3.2 Correlation analysis

**Figure 2:**
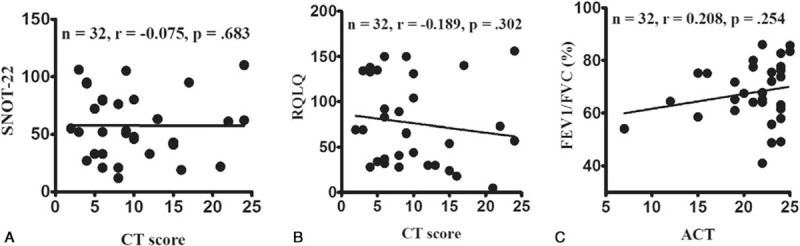
Patient-reported outcomes and objective clinical measurements are poorly correlated. Poor correlations were observed between the Sino-Nasal Outcome Test-22 (SNOT-22) and computed tomography (CT) scores (A), the Rhinoconjunctivitis Quality of Life Questionnaire (RQLQ) and CT scores (B), and the Asthma Control Test (ACT) and forced expiratory volume in 1 s (FEV_1_) values (C).

**Figure 3:**
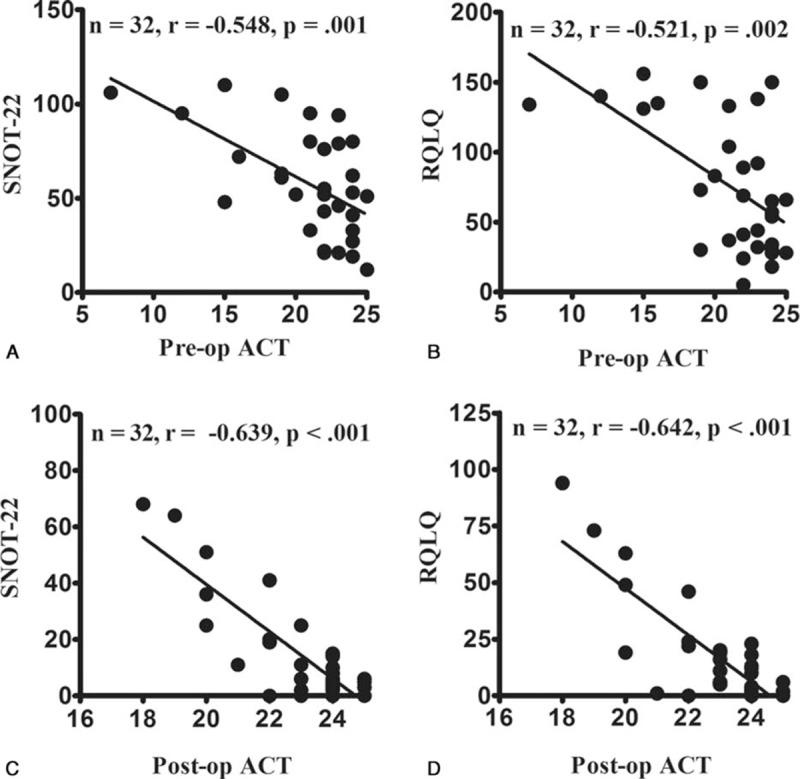
Correlations among the preoperative and 3-month postoperative scores. There were significant preoperative (pre-op) and 3-month postoperative (post-op) correlations between the Asthma Control Test (ACT) and Sino-Nasal Outcome Test-22 (SNOT-22) values (A and C), and between the ACT and Rhinoconjunctivitis Quality of Life Questionnaire (RQLQ) values (B and D).

**Figure 4:**
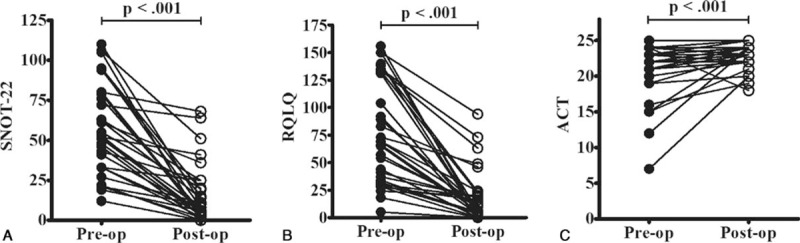
Nasal surgery improved sinonasal symptoms (A and B) and asthma control (C). ACT = Asthma Control Test, post-op = postoperative, pre-op = preoperative, RQLQ = Rhinoconjunctivitis Quality of Life Questionnaire, SNOT-22 = Sino-Nasal Outcome Test-22.

**Figure 5:**
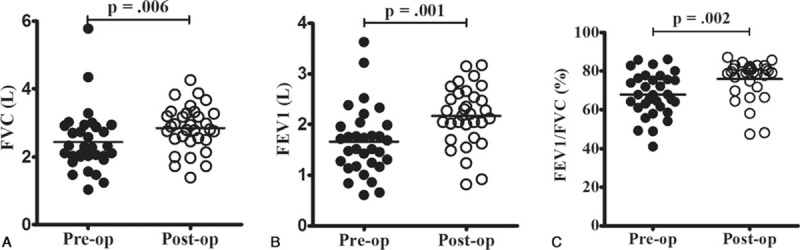
Compared to the preoperative (pre-op) pulmonary function test results, the postoperative (post-op) forced vital capacity (FVC) (A), forced expiratory volume in 1 s (FEV_1_) (B), and FEV_1_/FVC ratio (C) were all significantly improved.

When we analyzed the correlations between the questionnaire results and CT scores, we observed a poor correlation between the SNOT-22 and CT scores, as well as between the RQLQ and CT scores. Similarly, there was a poor correlation between the ACT and FEV_1_ results (*P* > 0.05) (Fig. 2). However, significant correlations were observed between the ACT and SNOT-22 results, as well as between the ACT and RQLQ results, at the preoperative and 3-month postoperative assessments (*P* < 0.01) (Fig. 3). Nasal surgery improved the sinonasal symptoms and asthma control (*P* < 0.01) (Fig. 4 and Table 2), and the postoperative FVC, FEV_1_, and FEV_1_/FVC values were all significantly better than the preoperative values (*P* < 0.01) (Fig. 5).

**Table 2:**
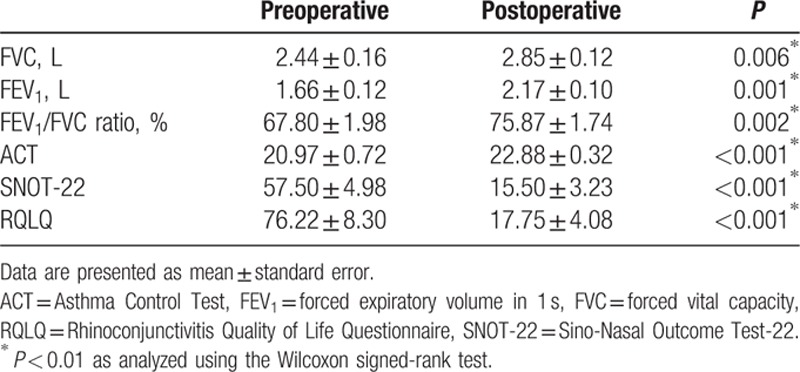
Preoperative and postoperative pulmonary functions and quality of life.

## 4 Discussion

To the best of our knowledge, this is the first study to focus on the link between CRS and asthma based on disease-specific QoL. In the present study, we used questionnaires to evaluate the symptoms of patients with asthma and CRS (who had failed medical treatment) before and after undergoing sinus surgery. Our results indicate that increasingly severe sinonasal symptoms of CRS were associated with poorly controlled asthma. Furthermore, nasal surgery improved the sinonasal symptoms, asthma control, and pulmonary function test results. Therefore, our findings appear to indicate that asthma stability is closely related to CRS control.

The airway is a continuous structure that extends from the nose to the alveolar units of the lung. The mucosal surfaces of the upper and lower airway are constantly exposed to the outside world, and inflammation may be induced by simultaneous exposure to various irritants, allergens, microorganisms, and other environmental factors (e.g., smoking). In addition, lower airway inflammation may be triggered through a systemic response or changes in the inspired air (e.g., dry, cold, and nitric oxide-depleted air) that are caused by nasal obstruction or aspiration of inflammatory sinus secretions into the lower airways.^[6]^ Therefore, although asthma and CRS are seemingly 2 distinct diseases, we believe that they should be viewed and treated as common airway diseases.

Interestingly, our results revealed that there were poor correlations between the SNOT-22 and CT scores, RQLQ and CT scores, and ACT and FEV_1_ values. These findings indicate that results from patient-reported questionnaires and objective clinical measurements (e.g., CT score and FEV_1_) are poorly correlated. In this context, patient-reported outcomes reflect the patient's dynamic experiences and effects of symptoms during a 4-week period, while the objective measurements are a static measure of disease severity at a single point in time. Thus, QoL instruments have an irreplaceable role in the assessment of disease severity, which is why the concept of asthma severity was replaced by the concept of asthma control (the main outcome measure that has been used in recent years).^[29,32]^ The ACT is another tool that is frequently used in clinical practice.^[22,23]^ Moreover, numerous studies have proposed that QoL instruments are a good tool for evaluating CRS severity and treatment outcomes (including after surgery).^[25–27]^

The topic of whether subjective measures of sinonasal disease can predict disease severity (as assessed using CT) has been explored in the literature. For example, a study by Nair^[33]^ revealed that symptomatic severity was correlated with specific sinus involvement that was confirmed using CT. Nevertheless, similar to our findings, many other studies have found that radiological CRS severity was not correlated with many self-reported QoL measures, such as SNOT-22 score, in the general population of patients with CRS, although there may be some correlation in certain subgroups, such as patients with CRS who have nasal polyposis or who smoke.^[34,35]^

Most patients in this study underwent nasal surgery when they exhibited relatively stable asthma control (average preoperative ACT score of 20.97), which may explain why most patients exhibited limited ACT score improvement. However, good ACT improvements were observed in patients with poor preoperative asthma control, and these patients also exhibited high CRS symptom scores. Although a large body of evidence from clinical epidemiology, pathophysiology, histology, and treatment outcomes has revealed a correlation between asthma and CRS with nasal polyps (CRSwNP),^[36–38]^ the preoperative ACT scores (22, 24, 15, 19, 19, and 22), postoperative ACT scores (24, 24, 21, 20, 23, and 23), and ACT improvement (2, 0, 6, 1, 4, and 1) from the 6 patients with CRSwNP were not significantly different from that of the patients with CRS without nasal polyps (CRSsNP). Unfortunately, the small sample size makes it difficult to analyze association between asthma and CRSwNP, and large-scale studies with subgroup analyses are needed to clarify the link between the different phenotypes of CRS and asthma.

This study has several limitations that warrant consideration. First, we did not compare the 3 questionnaires among patients who were not undergoing nasal surgery, and it would be useful to have a control group for the analyses. However, it is difficult to recruit appropriate and matched control patients, because the diverse triggers of asthma and CRS exacerbation may affect the outcome measures. Therefore, we performed analyses that revealed a dynamic correlation between CRS symptoms scores and asthma control before and after nasal surgery in a cohort of patients with CRS and asthma. Second, studies of patient-reported measures are susceptible to self-reported biases, such as changes in internal standards, changes in priorities, and changes in the interpretation of a given instrument. Thus, these biases may be present in our findings, although previous studies have validated the comparison of ACT, RQLQ, and SNOT-22 responses to quantify changes in symptoms and dysfunctions.^[21,22,24]^ Third, we only included patients with asthma who were undergoing sinus surgery for CRS, and these patients had relatively stable asthma control, which allowed them to undergo anesthesia and surgery. Fourth, our sample size was insufficient to perform disease subgroup analyses (e.g., of patients with CRSwNP or CRSsNP), and our follow-up interval was too short to evaluate longitudinal changes. Therefore, further large long-term studies are needed to validate our findings regarding the link between CRS and asthma based on patient-reported QoL, and to determine whether they can be observed in patients with unstable asthma.

## 5 Conclusion

We found that increasingly severe CRS symptoms were associated with poor asthma control, based on the responses to our CRS- and asthma-specific questionnaires. Furthermore, nasal surgery improved the sinonasal symptoms, asthma control, and pulmonary function test results. Therefore, we conclude that asthma and CRS should be considered and treated as common airway diseases.

## Acknowledgment

The authors thank Ms. Meng-Chieh Tsai for her contributions.
